# Analysis of predictors of mortality after surgical and non-surgical management in proximal humerus fractures

**DOI:** 10.1186/s10195-021-00606-7

**Published:** 2021-11-03

**Authors:** Alejandro Garcia-Reza, Diego Matias Dominguez-Prado, Constantino Iglesias-Nuñez, Lucia Alvarez-Alvarez, Beatriz Hernandez-Gonzalez, Patricia Balvis-Balvis, Daniel Fernandez-Fernandez, Manuel Castro-Menendez

**Affiliations:** 1 Department of Orthopaedic Surgery and Traumatology, Complexo Hospitalario Universitario de Vigo (Pontevedra), Estrada de Clara Campoamor, 341, 36213 Vigo, Pontevedra Spain; 2 Hospital Álvaro Cunqueiro, Estrada de Clara Campoamor, 341, 36212 Vigo, Pontevedra Spain

**Keywords:** Humerus, Fractures, Mortality, Comorbidities, Charlson comorbidity index

## Abstract

**Background:**

Proximal humerus fractures are one of the main osteoporotic fractures. Choosing between conservative or surgical treatment is a controversial topic in the literature, as is the functional impact. The main aim of our study was to analyse whether patient comorbidities should influence the final therapeutic decision for these fractures.

**Material and methods:**

We collected data from 638 patients with proximal humerus fractures. The main variable collected was exitus. We also collected the following data: age, gender, type of fracture, laterality, type of treatment, production mechanism, comorbidities and the Charlson comorbidity index (CCI) for each patient. The therapeutic indication used the criteria established by the Upper Limb Unit in our centre. We performed chi-square tests, Fischer’s exact tests and Student’s *t*-tests to compare the variables. We used the Kaplan–Meier method to analyse both the overall and disease-specific survival rates. We employed the Cox regression model to analyse factors associated with mortality.

**Results:**

Patients with a CCI greater than 5 showed greater mortality (HR  = 3.83; *p*  < 0.001) than those with a CCI lower than 5. Within the patients who underwent surgery, those with a CCI higher than 5 had an increased mortality rate (HR  = 22.6; *p* < 0.001) compared with those with a CCI lower than 5. Within the patients who received conservative treatment, those with a CCI over 5 showed greater mortality (HR  = 3.64; *p*  < 0.001) than those with a CCI under 5.

**Conclusions:**

Patients with proximal humerus fractures and associated comorbidities (CCI > 5) presented higher mortality than healthier patients. This mortality risk was greater in patients with comorbidities if surgical treatment was indicated rather than conservative treatment. Patient’s comorbidities should be a fundamental parameter when planning the therapeutic strategy.

**Level of evidence:**

Level 3.

## Introduction

Proximal humerus fractures (PHF), after proximal femur and distal radial fractures, represent the third most common fracture in patients over the age of 65 years [1]. They account for 5–6% of all fractures [2, 3], and are more frequent in women.

The incidence of PHFs increases with the patient’s age [4, 5]. Furthermore, they are one of the main osteoporotic fractures [6, 7]. In fact, it has been observed that an increase in the risk of falls is accompanied by a higher rate of hip fractures and PHFs [8] and increasing numbers of surgical treatments of these fractures have been observed over recent years [9]. This explains why studies of the functional impact and results of these operations have also increased.

Many articles have reviewed functional results after the management of this pathology [10–12], but only a few have analysed mortality related to PHFs and their different treatments. In addition, most of these articles have focused on inpatients or on patients undergoing surgery [13], and only a few studies have investigated both inpatients and outpatients [4, 14]. Neuhaus et al. [15] noted that factors such as aging, heart failure or chronic alcoholism are associated with adverse events in those patients hospitalised due to PHFs, and that intubation, ischemic heart disease or malignant disease are associated with in-hospital mortality. Fernández-Cortiñas et al. [16] noted that patients diagnosed with PHF and comorbidities, specifically those with a CCI greater than 5, have a significantly higher mortality rate than patients with lower CCI.

The CCI, which consists of stratifying the overall mortality risk of patients according to 19 items relating to their comorbidities, is one of the most used mortality predictors [17]. Nowadays, it is starting to be used in trauma patients, [18–20] and particularly in patients with PHFs [21, 22].

The objective of this study was to evaluate the mortality risk in patients with PHF who underwent surgical or non-surgical treatment, while also considering the patient’s characteristics and comorbidities and the complexity of the fractures.

## Material and methods

We performed a retrospective observational study (Level of Evidence III), obtaining the data on all patients diagnosed with PHF and treated in our hospital over 3 years (from January 1st, 2016 to December 31st, 2018). Our hospital is a tertiary centre for trauma patients and provides care to a population of about 470,000 inhabitants, including both urban and rural areas. We obtained data from the patients’ electronic medical records. This ensured the traceability of all the care visits made by patients in the National Public Health System.

### Inclusion criteria

All patients who suffered a proximal humerus fracture and were diagnosed and treated in our centre in the study period.

### Exclusion criteria

Patients under the age of 18 years old, pregnant women, polytraumatized patients, patients with pathologic fractures and those who did not receive follow-up care in our centre.

### Variables

The main variable collected during the follow-up was exitus. Other variables that we collected were the patient’s age and gender. We also assessed the type of fracture, which was evaluated and classified radiologically by five independent observers (general orthopaedic surgeons), according to the classifications of Neer and of the Arbeitsgemeinschaft für Osteosynthesefragen/Orthopaedic Trauma Association (AO/OTA) [23, 24]. We took anteroposterior and modified transthoracic supine lateral view X-rays of all the included patients. We performed a CT scan only to plan the surgery. We recorded the fracture laterality and the production mechanism: high-energy (traffic accidents, falls from over 3 m high) or low-energy (minor trauma, falls from less than 3 m high).

In addition, we also recorded each patient’s comorbidities prior to fracture, including cardiovascular diseases, neurological and psychiatric disorders, diabetes mellitus, osteoporosis, alcohol and tobacco abuse, as well as other endocrine, rheumatic and neoplastic diseases. We also calculated the CCI. Finally, we also recorded the type of treatment we used; both conservative and surgical.

The average follow-up time was 30 months, with a minimum of 15 and a maximum of 50 months.

### Therapeutic decision

We selected the surgical treatment according to the criteria of the Upper Limb Unit of the Trauma and Orthopaedics service in our centre. These criteria are similar to those described by Fernández-Cortiñas et al. [16], indicating surgery for displaced fractures whose parts contact less than 50% and/or with a variation of normal cervico-diaphyseal angle of more than 40° and/or with various deformities. These patients underwent surgery under general anaesthesia and nerve blocks, either by open reduction and internal fixation with osteosuture, Kirschner needles, blocking plates or intramedullary nailing, or by reverse total shoulder arthroplasty. Five different shoulder surgeons performed the operations.

We indicated conservative treatment when these criteria were not met or when the patient could not be operated on, according to anaesthetic criteria, due to comorbidities. This treatment consisted of immobilisation with a sling with anti-rotation webbing for 3 weeks.

In addition, all patients from both groups followed a program of physiotherapy exercises consisting of pendular exercises from the first week, passive range-of-motion exercises without exceeding 90° flexion and abduction from the second week, and finally active and active-assisted exercises from the third week, according to tolerance.

We conducted this study according to the Good Clinical Practice guidelines and the principles of the Declaration of Helsinki. The study protocol and data registration from the clinical records of patients were approved by the local Ethics Committee.

### Statistical analysis

We conducted a descriptive analysis of the variables with frequencies (percentages) and measures of central tendency (mean and standard deviation). We performed chi-square tests, Fischer’s exact tests and Student’s *t*-tests to compare these variables between the different groups of patients. We used the Kaplan–Meier method to analyse both the overall and disease-specific survival rates, and the log-rank test to compare the survival rate between different groups. We employed the Cox regression model to analyse factors associated with mortality and expressed them as hazard ratios (HR) with 95% confidence intervals (CI). We considered differences with a *p* < 0.05 as statistically significant. We conducted the analyses using SPSS v24.0 (IBM^®^).

## Results

We reviewed a total of 638 patients that met all the inclusion criteria. The youngest patient was 18 years old and the oldest 101 years old, with a mean age of 70.4 ± 14.8 years. Three hundred and thirty-eight patients (53.3%) were over 70 years old. The average follow-up time was 30.2 ± 11.1 months. Four hundred and ninety-five patients (77.6%) were women. The humerus fracture was the result of a low-energy trauma in almost all patients (96.2%) (Table 1).

**Table 1:** Sociodemographic characteristics and differences between groups

	Total (*n* = 638)	Alive (*n* = 582)	Deceased (*n* = 56)	*p* value^a^
Gender				
Female	495 (77.6%)	446 (90.1%)	49 (9.9%)	0.063
Male	143 (22.4%)	136 (95.1%)	7 (4.9%)	
Age (years)				
18–70	300 (46.7%)	291 (97%)	9 (3%)	**0.0001**
> 70	338 (53.3%)	291 (86.1%)	47 (13.9%)	
Laterality				
Right	331 (51.9%)	303 (91.8%)	28 (8.2%)	0.77
Left	307 (48.1%)	279 (90.9%)	28 (9.1%)	
Type of trauma				
Low-energy	614 (96.2%)	560 (91.2%)	54 (8.8%)	0.987
High-energy	23 (3.6%)	21 (91.3%)	2 (8.7%)	
Season				
Autumn–Winter	313 (49.1%)	281 (89.8%)	32 (10.2%)	0.205
Spring–Summer	325 (50.9%)	301 (92.6%)	24 (7.4%)	
Neer classification				
Non-displaced and 2-parts	361 (56.6%)	322 (89.2%)	39 (10.8%)	**0.03**
3- or 4-part and fracture-dislocation	277 (43.4%)	260 (93.9%)	17 (6.1%)	
AO-OTA classification				
Type A	314 (49.2%)	282 (89.8%)	32 (10.2%)	0.453
Type B	275 (43.1%)	255 (92.7%)	20 (7.3%)	
Type C	49 (7.7%)	45 (91.8%)	4 (8.2%)	
Treatment				
Conservative	513 (80.4%)	464 (90.4%)	49 (9.6%)	0.187
Surgical	125 (19.6%)	118 (94.4%)	7 (5.6%)	
Locking plate	47 (37.6%)	46 (97.9%)	1 (2.1%)	
Endomedullary nail	16 (12.8%)	14 (87.5%)	2 (12.5%)	
Kirschner needle	9 (7.2%)	9 (100%)	0	
Reversed total arthroplasty	36 (28.8%)	33 (91.7%)	3 (8.3%)	
Others	17 (13.6%)	16 (94.1%)	1 (5.9%)	
Comorbidities				
Cardiovascular	329 (51.6%)	286 (86.9%)	43 (13.1%)	**0.0001**
Neurological-psychiatric	187 (29.4%)	156 (83.4%)	31 (16.6%)	**0.0001**
Smoking-alcoholic abuse	46 (7.2%)	41 (89.1%)	5 (10.9%)	0.603
Obesity	37 (5.8%)	33 (89.2%)	4 (10.8%)	0.652
Diabetes	109 (17.1%)	95 (87.2%)	14 (12.8%)	0.101
Osteoporosis	63 (9.9%)	60 (95.2%)	3 (4.8%)	0.235
Rheumatologic	44 (6.9%)	42 (95.5%)	2 (4.5%)	0.317
Endocrinologic	90 (14.1%)	79 (87.8%)	11 (12.2%)	0.213
Charlson comorbidity index				
CCI 0–5	524 (82.1%)	500 (95.4%)	24 (4.6%)	**0.0001**
CCI > 5	114 (17.9%)	82 (71.9%)	32 (28.1%)	
Mean	3.71 ± 3.85	3.49 ± 3.9	6 ± 2.31	**0.0001**^**b**^

Bold values with statistically significant differences (*p* < 0.05)

^a^*p *value for Chi-square test for comparison of proportions between groups

^b^*p* value for *t* Student test for comparison of means between groups

We found 331 fractures (51.9%) in the right proximal humerus. As regards classifications, the most frequent AO-OTA type [24] was type A (49.2%). We found 43.1% type B fractures, and 7.7% type C. According to the Neer classification [23], 2-part fractures were the most frequent (32.3%). Then, in order of frequency, we found 3-part fractures (30.9%), non-displaced fractures (24.3%), 4-part fractures (6.9%) and finally 5.6% of fractures occurring as proximal humerus fracture-dislocations.

Most patients (80.4%) received conservative treatment. Within the patients who underwent surgery (19.6%), the most common practice was osteosynthesis (76 patients, 66.7%), with locking plates being the most frequent (47 patients, 37.6%), followed by intramedullary nailing (16 patients, 12.8%). We treated a total of 36 patients (28.8%) with shoulder arthroplasty.

With regard to the comorbidities, we calculated the age-adjusted CCI for each patient. The average CCI was 3.71 ± 3.85. We stratified our sample into two groups: patients with a CCI between 0 and 5 (82.1%) and patients with a CCI higher than 5 (17.9%). In addition, 51.6% of patients had prior cardiovascular diseases, 29.4% a psychiatric or neurological disorder, 5.8% were obese, 17.1% suffered from diabetes mellitus, 9.9% were undergoing treatment for osteoporosis, 6.9% had rheumatic diseases and 14.1% had endocrine diseases.

As regards mortality (Table 2), we recorded a total of 56 deaths (8.8%) up to the final date of data collection. The median overall survival was 46.57 months (95% CI 45.71–47.43). We saw greater mortality [HR  = 3.83 (95% CI: 2.15–6.81); *p*  <  0.0001] in patients with a CCI higher than 5. These patients presented a median survival time of 39.34 months (95% CI 36.22–42.47), which was lower than the average survival of patients with CCI under 5, which was 48.16 months (95% CI 47.44–48.88).

**Table 2:** Mortality risk factors in multivariate Cox regression survival analysis

	HR [CI 95%]	*p* value
Age > 70 years old	2.84 [1.31–6.15]	0.008
3- or 4-part and fracture-dislocation	0.5 [0.281–0.89]	0.018
Charlson CCI > 5	3.83 [2.15–6.81]	0.001
Neurological-psychiatric comorbidities	2.41 [1.40–4.12]	0.0001

*HR* hazard ratio; *CI 95%* confidence interval 95%

There were no significant differences in the mortality risk (*p*  =  0.221) between patients that received conservative treatment (9.6% exitus) as opposed to those that received surgical treatment (5.6% exitus). In the subgroup analysis, we saw that, within the group of patients that received conservative treatment, those with a CCI higher than 5 had a higher risk of mortality [HR  = 3.64 (95% CI 1.97–6.76); *p*  < 0.0001] than patients with a CCI under 5. This increase in the mortality risk in patients with more comorbidities with respect to the healthier ones turned out to be much higher in the group of patients that received surgical treatment [HR  =  22.6 (95% CI 3.93–129.95); *p*  < 0.0001].

In the group of patients with a CCI higher than 5, the average survival was 28.51 months (95% CI 26.09–31) when conservative treatment was decided upon, but when the decision was surgical treatment, the average survival was 22.2 months (95% CI 14–29.67). There were no differences in the mortality risk [HR  = 0.56 (95% CI 0.13–2.38), *p*  =  0.44].

**Fig. 1:**
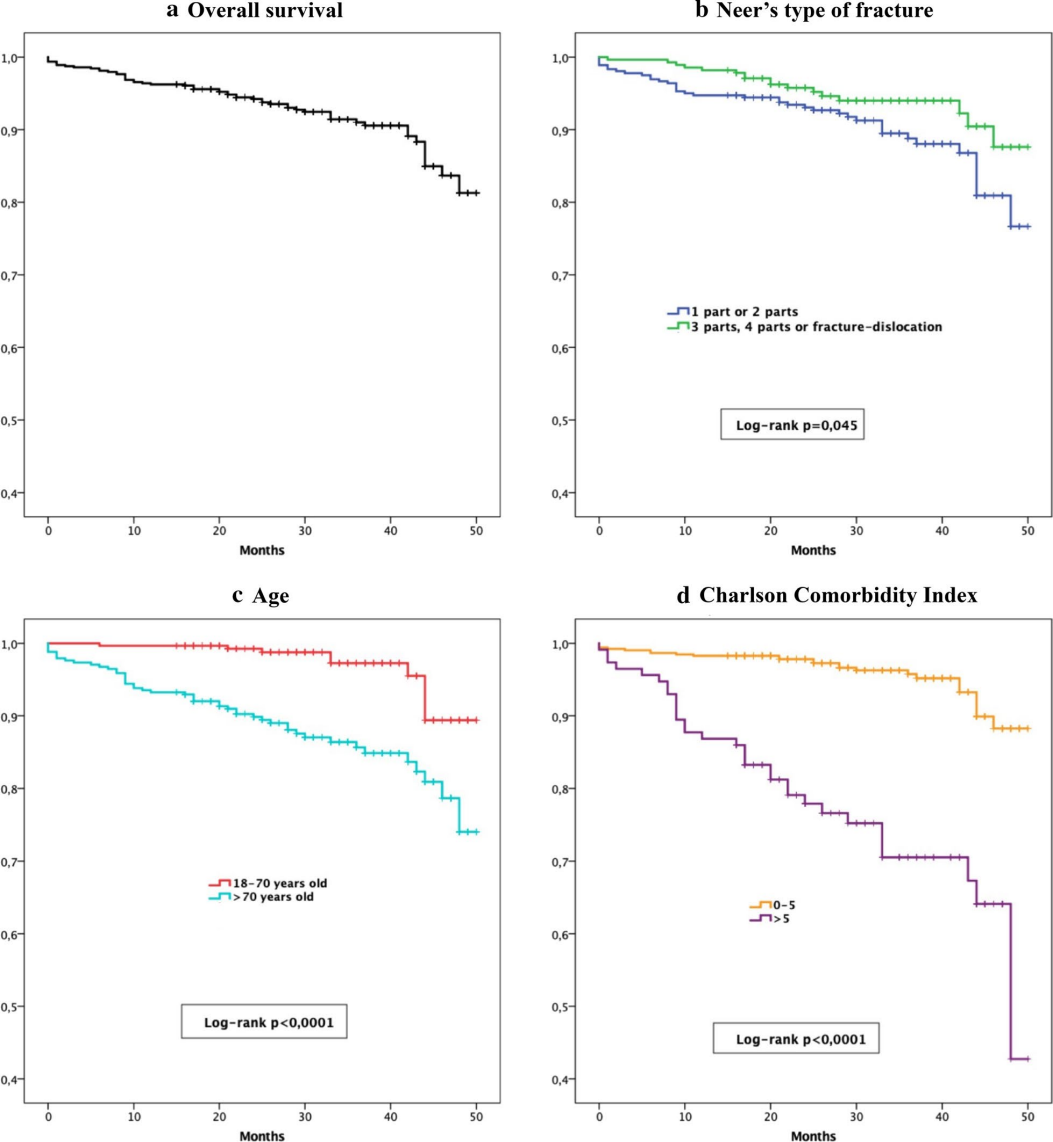
The graphs show Kaplan–Meier survival curves for **A** overall survival, **B** type of fracture, **C** age and **D** Charlson comorbidity index

We noted that patients over 70 years old had a higher mortality risk [HR  = 2.84 (95% CI 1.31–6.15); *p*  = 0.008] than those aged 70 years or younger (Fig. 1). The median survival of patients over 70 years old was 44.58 months (95% CI 43.14–46.03), while the others had a median survival time of 48.85 months (95% CI 48.12–49.58).

Regarding the analysis of mortality risk of the different comorbidities, the multivariate Cox regression model, adjusted for age, type of fracture and CCI only showed statistical significance in the mortality risk of patients with neurological or psychiatric disorders [HR  = 2.41 (95% CI 1.40–4.12); *p*  < 0.0001]. There were no statistically significant differences for mortality risk in the other comorbidities studied.

We divided the patients into two groups according to the Neer classification. The first group of less complex fractures included non-displaced fractures and 2-part fractures. The group of greater complexity included 3- and 4-part fractures and fracture-dislocations. We saw that patients with 3- and 4-part fractures and fracture-dislocations had a lower mortality risk [HR  = 0.5 (95% CI 0.281–0.89); *p*  =  0.018] than patients with non-displaced fractures and 2-part fractures. More complex fractures had an average survival time of 47.69 months (95% CI 46.63–48.75) while most simple fractures had an average survival time of 45.73 months (95% CI 44.46–46.99). In the subgroup analysis, this effect was not statistically significant in patients undergoing surgery (*p * = 0.596), but it was in patients receiving conservative treatment (*p*  = 0.026).

We found no statistically significant differences within the surgically treated group for exitus (*p*  =  0.794), among the osteosynthesis group (4.2%) or the arthroplasty group (8.3%). There were no differences in the mortality risk either [HR  = 2.048 (95% CI 0.41–10.18), *p*  = 0.381]. Analysing the age distribution in these two groups, we detected that the group treated with arthroplasty presented a significantly higher proportion of patients older than 70 years than the group treated with osteosynthesis (66.7% versus 38.5%, *p*  < 0.0001). Likewise, as regards fracture complexity, we noted that the arthroplasty group had a significantly higher proportion of 3- and 4-part fractures and fracture-dislocations than the osteosynthesis group (88.9% compared with 54.2%, *p* < 0.0001).

## Discussion

Mortality in PHFs has been significantly associated with old age, cardiovascular problems and even alcohol abuse, which alters the inflammatory response to injury [25]. In our study, we observed that patients over the age of 70 years had a higher risk of mortality (HR  = 2.84) than younger patients. However, we did not find any relationship between mortality and diabetes mellitus or alcohol abuse. Nevertheless, we were limited by the fact that alcohol abuse is complex to establish, since it is not usually recognised or is not recorded exhaustively in the electronic medical records. In our sample, we recorded alcohol abuse along with tobacco consumption, and obtained a prevalence of 7.2%.

As regards the treatment for PHFs, a recent Cochrane Review (2015) [26] provides high or moderate quality evidence that, when compared with non-surgical treatment, surgery does not give better results 1 or 2 years after the injury in patients with displaced PHFs, including the neck of the humerus, and these fractures are likely to lead to greater subsequent need for surgery. Neuhaus et al. [15] note that open reduction and internal fixation of these fractures is associated with a high risk of adverse events and increased mortality compared with conservative treatment, and add that surgical treatment involves significant short-term risks that must be taken into account when making decisions, especially with elderly patients. In the field of arthroplasty, Rotman et al. [27] compared conservative treatment with reverse total shoulder arthroplasty in patients with complex displaced PHF. They found no significant differences in mortality over a year, although they reported a trend that shows lower mortality with arthroplasty, especially in men.

Like most authors, we did not find statistically significant differences in the mortality risk between those patients who received conservative treatment and those who underwent surgery. Dabija et al. [28] noted that arthroplasty has a greater need for surgical reoperation compared with conservative treatment and osteosynthesis. Reinier-Beks et al. [29] concluded that there are more complications requiring reoperation after surgery of a displaced fracture of the humerus, without having improved functional outcomes. They do not, therefore, recommend surgery in patients over 65 years with displaced PHF. Similarly, the ProFHER randomised clinical trial by Rangan et al. [30] found no significant differences in functionality between conservative and surgical treatment in patients with displaced fractures of the surgical neck. In contrast, Lander et al. [31] recently concluded that there is lower mortality in patients with PHF undergoing surgery, although their study included only patients who were hospitalised and were over 60 years old.

Analysing those patients undergoing surgery, we found no significant differences in mortality between patients undergoing osteosynthesis (plates, intramedullary nailing, Kirschner wire) or reverse total shoulder arthroplasty. The group that was treated with arthroplasty had statistically significantly more patients older than 70 years (66.7%), and the vast majority (88.9%) had 3- or 4-part fractures or fracture-dislocations. We have not found other studies that compare mortality directly between both types of surgery in patients with PHF. Only Dixit et al. [32] observed no significant difference as regards mortality between osteosynthesis and arthroplasty in open PHF.

In assessing the CCI in our study, we observed that patients with a score above 5 had a significantly higher mortality risk (HR  = 3.83) than patients with a lower score. This finding is in line with Myeroff et al. [33], who reported that an increase of one point in the CCI is associated with an increase in mortality of up to 40% in patients with PHFs. Fernández-Cortiñas al. [25] showed that patients with multiple comorbidities (high CCI) who underwent surgery had a higher risk of mortality (HR  = 6.9) than patients in the same group who underwent conservative treatment (HR  = 4.1).

In addition, in a recent meta-analysis with more than 70,000 patients, Floyd et al. [34] noted that high rates of surgery are associated with an increased mortality risk in the first year, and this is especially pronounced in aged cohorts and in those with many comorbidities. In our case we observed a greater difference. We noted that, when receiving surgical treatment, patients with multipathological problems (CCI  > 5) had a much higher mortality risk (HR  = 22.6) than those who had received conservative treatment (HR  = 3.66), compared with the group of healthier patients (CCI  < 5). These findings suggest that surgical treatment in patients with multipathological problems increases mortality considerably and strengthens the theory that the CCI is a valid predictor tool of mortality in patients with PHFs [22]. Therefore, we recommend considering the patient’s preoperative comorbidities as a fundamental parameter to decide between one treatment or the other, in the same way as other parameters such as the type of fracture, the extension and angulation, the functional state of the patient or other health considerations.

Both the AO-OTA and the Neer classifications [23, 24] remain complex and even more advanced imaging systems have not made it possible to improve interobserver reproducibility [35]. Nevertheless, it has been pointed out that the Neer classification is reproducible enough to allow comparisons between different studies [36].

The degree of complexity of PHFs, classified according to Neer, has been valued on many occasions in relation to functional results according to types of treatment, without finding clear statistically significant results that demonstrate the superiority of surgical over conservative treatment in 3- or 4-part fractures [11, 29]. With regards to mortality, Myeroff et al. [33] noted that, although it is related to the hospitalisation of the patient, there is no significant relationship between mortality and the type of fracture according to the Neer classification. Nonetheless, they associated this absence of significance with the relative infrequency of more complex fractures. They studied each type of fracture separately, without grouping them. However, to reduce interobserver variability, we sorted the fractures in our sample into two groups according to the Neer classification: the group of less complex fractures (non-displaced fractures or 2-part fractures) and the group of greater complexity (3- or 4-part fractures and fracture-dislocations). We found that the more complex fractures had a statistically significantly lower mortality risk than simpler fractures (HR  = 0.5), a protective effect that was also maintained in the subgroup analysis of patients who received conservative treatment. The median survival time was 45.73 months in the group with less complex fractures and 47.69 months in the group with greater complexity. This division into two groups for analysis was also followed by Rangan et al. [30] in their ProFHER multicentre randomised clinical trial, with a total of 250 patients. They compared equally non-displaced fractures or 2-part fractures against 3- or 4-part fractures, although they did not find statistically significant differences.

This protective effect that we found in our study was not significantly influenced by either age or by differences in the CCI between both comparison groups (Table 2). Since we included fracture-dislocations in the group of greater complexity, unlike in the other studies [30, 33], we think that these fractures may be acting as a confounding factor since we observed 0% mortality in our sample, in spite of the fact that they accounted for 5.6% (36 patients) of the sample. However, when removing these fractures from the survival analysis, the group of more complex fractures still presented a statistically significant protective effect compared with the group with fractures that are a priori simpler.

It is possible that, in our sample, the most complex fractures occurred in more active patients, who are usually associated with faster recovery and greater overall survival. However, we do not have the data necessary to be able to make such a conclusion, so these statements must be analysed with caution. Furthermore, more studies would be needed to corroborate this hypothesis.

Our study suffers the limitations of being a retrospective observational study. We did not include patients with follow-up care in the private healthcare sector, although the total number of patients that could be added to the sample would be very small, since the vast majority of our society receives treatment and follow-up care for these fractures in the National Public Healthcare System. Some variables, such as alcohol abuse, are difficult to collect and are often overlooked in a general trauma clinical interview. In addition, another variable, such as the Neer classification, suffers great inter- and intra-observer variability. Nevertheless, it is generally the most used classification and allows comparison between different working groups.

As a strength of our study, it should be noted that we analysed data from a total of 638 patients, a larger sample than that of many of the studies published to date [11, 16, 29, 33]. Furthermore, our study also encompasses the totality of PHFs that occurred in the healthcare area of a tertiary hospital for a period of 3 years. In addition, thanks to the full health coverage of our population in the public system and the global nature of the system of electronic medical records in all the healthcare centres in the region, we had access to the data of all the health care records to assess the comorbidities and events studied, as well as data on scheduled medications and follow-up visits. This permitted total and comprehensive traceability of all patients included in the study. We were able to precisely identify those patients surgically treated and their postoperative care as well as those patients who received conservative treatment on an outpatient basis with its subsequent evolution. In addition, the mortality event is electronically recorded on the same date.

## Conclusions

There are no differences in mortality in the medium-long term between surgical and conservative treatments in patients with PHF. However, those patients with PHFs and associated comorbidities (CCI  > 5) show a higher mortality risk than healthier patients. This mortality risk is greater in patients with comorbidities when surgical treatment is chosen instead of conservative treatment. A patient’s comorbidities must be a fundamental parameter when planning the therapeutic strategy.

More complex PHFs do not imply greater mortality and, in fact, they have a lower mortality risk with respect to less complex fractures; however, this fact should be confirmed with subsequent studies.

## Data Availability

The datasets used and/or analysed during the current study are available from the corresponding author on reasonable request.
